# Paediatric fractures in a sub-saharan tertiary care center: a cohort analysis of demographic characteristics, clinical presentation, therapeutic patterns and outcomes

**DOI:** 10.11604/pamj.2017.27.46.11485

**Published:** 2017-05-18

**Authors:** Marc Leroy Guifo, Joel Noutakdie Tochie, Blondel Nana Oumarou, Jean Roger Moulion Tapouh, Aristide Guy bang, Aurelien Ndoumbe, Bonaventure Jemea, Maurice Aurelien Sosso

**Affiliations:** 1 Department of Surgery, University Teaching Hospital of Yaoundé, Yaoundé, Cameroon; 2 Department of Surgery and specialties, Faculty of Medicine and Biomedical Sciences, University of Yaoundé, Yaoundé, Cameroon; 3 National Social Insurance Fund Health Center of Yaoundé, Yaoundé, Cameroon; 4 Department of Radiology and Medical Imaging, University Teaching Hospital of Yaoundé, Yaoundé, Cameroon; 5 Faculty of Medicine and Pharmaceutical Sciences, University of Douala, Douala, Cameroon

**Keywords:** Paediatric fractures, clinical presentation, manipulation under anaesthesia

## Abstract

**Introduction:**

Paediatric fractures are often of good prognosis due to auto-correction of insufficient fracture reduction by bone remodeling. In sub-Saharan Africa, traditional healers are renowned for managing fractures and there is a neglect for specialized pediatric fracture care. We aimed to determine the demographic characteristics, clinical presentation, treatment patterns and outcomes of paediatric fractures in a tertiary health care centre in Yaoundé.

**Methods:**

We conducted a prospective cohort study of all consenting consecutive cases of fractures in patients younger than 16 years managed between January 2011 and June 2015 at the University Teaching Hospital, Cameroon. We analysed demographic data, injury characteristics, fracture patterns, treatment details, therapeutic challenges and outcome of treatment at 12 months of follow-up.

**Results:**

We enrolled 147 fractures from 145 children with a mean age of 7 years and male-to-female sex ratio of 2.5:1. The main mechanisms of injury were games (53%) and accidental falls (20.7%). Forearm fractures were the most common fractures (38%). The mainstay of management was non-operative in 130 (88.5%) fractures, with 29.3% manipulations under anesthesia and 17 (11.5%) open reductions with internal fixation. The most surgically reduced fractures were supracondylar humeral fractures. Major difficulties were long therapeutic delay, lack of diligent anaesthesia and the lack of fluoroscopy. The outcome of treatment was favorable in 146 (99.3%) paediatric fractures.

**Conclusion:**

With the growing population of sub-Saharan Africa and the objective of becoming an emergent region, public policies should match the technical realities.

## Introduction

Injuries represent a frequent cause of admission into pediatric orthopedic and traumatology departments. Fractures occur in about 25% of all injured children [1]. The mechanisms of these injuries vary from accidental falls during recreational activities to road traffic accidents. Non-operative management is the mainstay of treatment of paediatric fractures, with reported good outcomes owing to the active remodelling potential of children's periosteum which speeds up the fracture healing process [2, 3]. As such, paediatric fractures are increasingly being managed by non-orthopedic surgeons, including traditional healers in sub-Saharan Africa. Although less frequent, there exist some indications for surgical fixation of paediatric fractures, namely; open fractures, lateral condyle fractures of humerus, displaced supracondylar humeral fractures,femoralfractures in school aged children and the presence of associated injuries like head trauma or vascular lesion [1, 4]. The management of paediatric fractures in low-income countries is suboptimal, predisposing injured children to an increased risk of carrying physical disabilities and other complications into adulthood. We systematically applied the guidelines of the paediatric orthopaedic and traumatology department of Brugmann hospital (Hopital Universitaire des Enfants Reine Fabiola) in Brussels where we underwent a fellowship, in a bid to identify the therapeutic challenges to standard and safe management of paediatric fractures in our resource-limited setting and thereby propose local solutions to amend these guidelines to our specific environment.

## Methods

**Figure 1:**
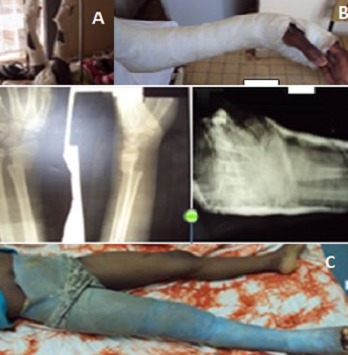
Different type of non-operative treatment; gallow traction on a locally made support, a BAB plaster and a single-leg spica cast (from top to bottom)

**Study design, setting and participants**: We carried out a prospective cohort study between the 1stJanuary 2011 to the 30thJune 2015 at the surgical department of Yaoundé University Teaching Hospital, Cameroon. All patients aged less than 16 years presenting with a fracture managed and follow-up for a minimum of one year in the aforementioned surgical department, were consecutively enrolled. Patients who could not be followed up till fracture consolidation, those whose parents did not consent for enrollment or who were first treated in another health center were excluded from the study. From the available literature, an interviewer administered pre-tested questionnaire was used to study the following variables; demographic data: age, sex, telephone number; the date of injury; the date of treatment; fractures characteristics: type of injured bone, type of injured bone segment, AO or Salter Harris classification where appropriate; type of treatment; the need for fracture reduction; type of anaesthesia technique used; and the outcome. The outcome was evaluated as poor if there was mal-union that could not be corrected by bone remodeling; acceptable when insufficient reduction was corrected by bone remodeling with no resultant functional impairment; and good when the reduction was anatomical.The following fracture types were considered as indications for open reduction and fixation; displaced supracondylar humeral fractures (Gartland Type III), lateral humeral condylar fractures, displaced forearm fractures or insufficient reduction, femoral fractures in skeletal matured children, opened Gustilo III fractures of any limb, multiple fractures and fractures associated with head injury. The type of treatment varied according to the bone location of the fracture and included therapeutic modalities such as Gallow traction, single leg spica and BAB plaster (Figure 1).

**Data analysis**: The data obtained were entered into Epi info 3.5.1 statistical software. Variables were presented in simple frequencies and means of numerical variables were reported. Patients lost to follow-up were excluded from the final analysis.

**Ethical considerations**: The Ethics Committee of the Yaoundé University Teaching Hospital, Cameroon approved the realization of this study. Parental consent was obtained for all pictures of paediatric fractures illustrated in this study.

## Results

**Demographic characteristics and clinical presentation**: A total of 145 children with 147 fractures were recorded during the study period. There were 104 (71.7%) males and 41(28.3%) females. The mean age of children was 7 years. The injured side was the right in 56 cases (39%) and left in 89 (61%). The time laps between injury and the initial consultation varied from 30 minutes to 120 days post injury. The mean delay from initial consultation to fracture management was 3 days. The most common mechanisms of injury were games in 77 cases (53.1%) follow by 30 (20.7%) accidental falls, 26 (17.9%) road traffic accidents, and 6 (4.1%) birth injuries as depicted in Table 1. With regards to fracture location, the forearm was the most involved with 55 (37%) cases follow by 37 (25.5%) humeral and 23 (16%) femoral fractures (Table 2).

**Table 1:** Distribution of patients according to the mechanism of injury

Mechanisms of Injury	Frequency (n=145)	Percentage (%)
Games	77	53.1
Accidental falls	30	20.7
Road traffic accidents	26	18.0
Birth injuries	06	4.1
Sport	01	0.7
Fight	01	0.7
Unknown	04	2.7

**Table 2:** Distribution of patients according to the fracture site

Fracture site	Frequency (n=147)	Percentage (%)
Forearm	55	37
Humerus	37	25.5
Clavicle	06	4.13
Femur	23	16
Leg	23	16
Ankle	03	02

**Figure 2:**
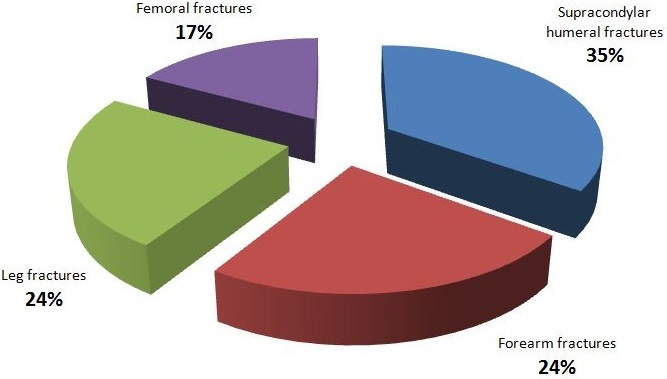
Distribution of surgically reduced fractures

**Figure 3:**
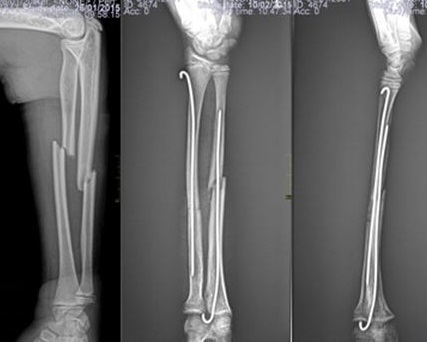
Case of forearm fracture treated by intramedullary pins

**Therapeutic patterns and challenges**: With respect to the treatment of the 147 fractures; 77 (52.4%) were managed by plaster cast immobilization without reduction, 10 (6.8%) were treated by traction followed by casting, 43 (29.3%) underwent closed reduction followed by plaster immobilization and 17 (11.5%) had surgical fixation. Three anesthesia techniques were used, namely; general anaesthesia with halogen volatile gases for open reduction and internal fixation procedures in 29 (48.34%) cases, general anesthesia with ketamine in 12 (20%) cases and local anesthesia by hematoma block with xylocaine in 19 (31.7%) cases. The reduction methods entailed 43 (29.3%) manipulations under anesthesia (MUA) and 17 (11.5%) open reductions with internal fixation. The most surgically reduced fractures were supracondylar humeral fractures (35%), followed by fractures of the forearm (23.5%) and leg (23.5%) as illustrated in Figure 2 and Figure 3.

**Figure 4:**
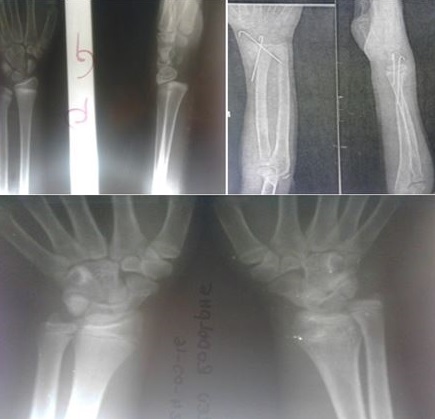
Epiphysiodesis complicating a Salter Harris type 1fracture of the distal radius

**Outcome of treatment**: Overall, the outcome of treatment was rated as good or acceptable in 146 (99.3%) paediatric fractures, except a poor outcome in a Salter Harris Type I fracture of the distal radius initially managed closed reduction, but presented 3 weeks later with unacceptable reduction requiring further management by open reduction and pin fixation. The postoperative outcome was complicated by epiphysiodesis (Figure 4).

## Discussion

In this cohort of 145 children with fractures, we noticed that despite the fact that non-operative treatment was more common with 59.2 % cases, there was a necessity of fracture reduction through MUA for 29.5 and open reduction and internal fixation in 11.5%. Our findings were consistent with previous local epidemiological concepts showcased by Mouafo et al [5] in 2011 about paediatric fractures namely; a higher incidence in male children,most injured children aged between six to eleven years, a predilection of fractures on the non-dominant limb and often affecting the upper limbs. It is worth noting that all the fractures were not seen by the surgical team since the attending physicians at the emergency department were not always instructed to call the latter and there was no prevailing mechanism to ensure that all injured children should have the right to specialized orthopedic care. Most of the fractures occurred during games (53.1%) and accidental falls (20.7%), explained by the fact that unlike adults, children do not possess matured cognitive and perceptuo-motor abilities to avoid accidental injuries [6, 7]. As such their physical strength outweighs judgment, and protective reflexes are not fully developed making them a high risk group for fractures [8]. Also, the high proportion of paediatric fractures resulting from games (53.1%) may equally reflect the absence of parental awareness or education and the tendency of children to play at home or in in-secured playing grounds unsupervised. The most common indication for surgical fixations were displaced supracondylar humeral fractures (35%), managed by open reduction and internal fixation. These specific fractures posed the therapeutic challenges ofvarus deformity,equally observed by Yotsuka et al [9]. Similarly to Newton et al [10], femoral fractures were associated with major therapeutic difficulties, namely; longer periods of immobilization under traction followed by casting in both pre-school and school aged children between 3 and 10 years, with resultant financial losses experienced by their caterers. Attempting to remedy this situation, Jaafar et al [11] obtained satisfactory results following immediate casting without traction for all children aged between one to three years. In our series, parents who could not financially withstand hospital stay under traction for a duration of one weeks and were discharged home earlier after being educated on how to cater for their child on zenith traction at home, as recommended by some authors [10]. Our surgical fixation methods comprised of kirchner pin for displaced supracondylar humeral fractures and forearm fractures; plate fixations for closed displaced humeral shaft, femoral and tibial fractures; and external fixations for opened Gustilo III fractures. Pin or plate fixations were exceptionally indicated for opened Gustilo II fractures, if these fractures were managed within 6 hours of injury. Although flexible intramedullary nailing has revolutionized the management of diaphyseal fractures in children before skeletal maturity [12–14], the unavailability of fluoroscopyin our health care setting significantly hindered its use. In a bid to solve this problem, Yaokreh et al [12] in Ivory Coast, obtained satisfactory results following flexible intramedullary nailing after opening of the fracture site in the absence of a fluoroscope. This may be an alternative fixation technique, provided the procedure is well known by the surgical team.

We observed a mean therapeutic delay of three days from the initial presentation to the fracture management in our health care setting. Similar observations were made by Olney et al [15], owing to priority care given to more life threatening conditions. Our therapeutic delay is explained by a deficiency in health care setting organization and the lack of skills in the use of rapid and short acting anesthetic protocols like nitrous oxide for fracture reductions. In other settings, alternative anesthesia techniques used in pediatric fracture reduction include sedation with ketamine or halogen volatile gases and hematoma block with xylocaine. In our study, we used all these anesthesia techniques except sedation with Nitrous oxide which has been portrayed to be less invasive, safe and does not require skilled labour for its administration. However, the use of Nitrous oxide is often entails adjuvant anesthesia with hematoma block in some orthopedic and traumatology departments because of the weak analgesic properties of the former [16–18]. In settings where Nitrous oxide is available we can use the C-arm to inject the xylocaine solution precisely in the fracture site because one of the major inconvenient of local anesthesia with xylocaine is inaccurate injection and consequent pain during manipulations. Overall, we highlight significant therapeutic difficulties encountered in paediatric trauma care in low-income countries. With increase demand in health care, performance, safety and quality control are becoming part of health care systems evaluation. Therefore health professional will no longer morally and legally afford to practice in suboptimal conditions. Solutions for resource-limited settings may include measures which reduce hospital stay like home traction for toddlers and a brief period of traction followed by casting in school aged children. Means for flexible intramedullary nailing and image intensifier fluoroscopy, should be put at the disposal of centers managing paediatric fractures. Also, implementation of national health insurance may ensure that injured children are being given the appropriate treatment and help resolve parental financial constraints. The main drawback of our study is its single study setting. As such, our findings may be generalized to the entire nation with caution. However, based on an acceptable sample size (n=145) of well followed-up patients, we have used a cohort design to provide a contribution of level II scientific evidence to the scarcity of data on the demographic characteristics, clinical presentation, therapeutic patterns and outcomes of paediatric fractures in a sub-Saharan African setting.

## Conclusion

Our findings suggest that paediatric fractures are frequent causes of admissions to our University Teaching Hospital. Although favourable outcomes were obtained from both operative and non-operative management, we encountered major therapeutic challenges namely; long therapeutic delay, lack of diligent anaesthesia techniques for closed reduction procedures, unsatisfied parents by prolonged immobilization of children on traction and casts and the lack of fluoroscopy. Due to this high frequency of fractures in the pediatric population and the management challenges akin to resource-limited settings, we highlight the urgent need of improving paediatric orthopedic care and equipping centers managing paediatric fractures. The benefits of reducing the long term complications of poorly managed paediatric fractures cannot be overemphasised in a setting already faced with increasing physical handicap burden in children and adults from non-traumatic origin.

### What is known about this topic

- Fractures in pediatric population are frequent admission reason in hospital;
- Despite the favorable prognosis that is observe and concurrent orthopedic management of these cases, some localization and presentation are recognize surgical indications and therefore need more specialize care.

### What this study adds

- This study attempts to estimate the burden of fracture management in a tertiary care facility in our environment and recognize that despite the majority of fracture in pediatric population do not need surgical treatment, 30% of non operative management need manipulation under anesthesia;
- In order to avoid inappropriate care that could lead to functional handicap in adulthood, health personnel need to be sensitized and train to recognize difficult cases and provide appropriate treatment.

## Competing interests

The authors declare no competing interest.
